# Interleukin-27 in T Cell Immunity

**DOI:** 10.3390/ijms16022851

**Published:** 2015-01-27

**Authors:** Yukiko Iwasaki, Keishi Fujio, Tomohisa Okamura, Kazuhiko Yamamoto

**Affiliations:** 1 Department of Allergy and Rheumatology, Graduate School of Medicine, the University of Tokyo, 7-3-1 Hongo, Bunkyo-ku, Tokyo 113-8655, Japan; E-Mails: yunyan-todai@umin.ac.jp (Y.I.); tomohisa-tky@umin.ac.jp (T.O.); yamamoto-tky@umin.ac.jp (K.Y.); 2 Max Planck-The University of Tokyo Center for Integrative Inflammology, the University of Tokyo, 4-6-1 Komaba, Meguro-ku, Tokyo 153-8505, Japan

**Keywords:** IL-27, IL-27p28, Ebi3, T cell subset

## Abstract

Interleukin (IL)-27, a member of IL-12/IL-23 heterodimeric family of cytokines, has pleiotropic properties that can enhance or limit immune responses. IL-27 acts on various cell types, including T cells, B cells, macrophages, dendritic cells, natural killer (NK) cells and non-hematopoietic cells. Intensive studies have been conducted especially on T cells, revealing that various subsets of T cells respond uniquely to IL-27. IL-27 induces expansion of Th1 cells by activating signal transducer and activator of transcription (STAT) 1-mediated T-bet signaling pathway. On the other hand, IL-27 suppresses immune responses through inhibition of the development of T helper (Th) 17 cells and induction of IL-10 production in a STAT1- and STAT3-dependent manner. IL-27 is a potentially promising cytokine for therapeutic approaches on various human diseases. Here, we provide an overview of the biology of IL-27 related to T cell subsets, its structure, and production mechanism.

## 1. Introduction

Interleukin (IL)-27 is a heterodimeric cytokine composed of Epstein-Barr virus-induced gene 3 (Ebi3) and IL-27p28, belong to the IL-6/IL-12 family cytokines. Its receptor is composed of gp130 and IL-27 receptor α chain (IL-27Rα, also known as WSX1 or TCCR) that activates Janus kinase (JAK)/signal transducer and activator of transcription (STAT) pathway and mitogen activated protein kinase (MAPK) pathway.

At first, IL-27 was recognized as an inflammatory cytokine, depending on the fact that IL-27Rα deficient (IL-27Rα KO) mice showed susceptibility to *Listeria monocytogens* or *Leishmania major* infection and that IL-27 supported proliferation and interferon (IFN)-γ production in CD4^+^ T cells [1]. It was also reported that IL-27 induces the expansion of type 1 helper T (Th1) cells by activating the STAT1-mediated T-box expressed in T cells (T-bet) pathway [2]. In relation to CD4^+^CD25^+^ regulatory T cell (Treg), which characteristically express the transcription factor forkhead box protein p3 (Foxp3), Cox *et al.* reported that IL-27 suppresses differentiation of inducible Treg under IL-2 and transforming growth factor (TGF)-β stimulation and that Foxp3 expression is enhanced by IL-27Rα deficiency in the mouse colitis model induced by transfer of naïve CD4^+^ T cells [3]. It was also reported that severe systemic inflammation occurs in IL-27-transgenic mice because of the impaired development of Foxp3^+^ Treg due to reduced IL-2 production [4]. However, the number and percentage of Foxp3^+^ Treg shows no remarkable change in IL-27- or IL-27Rα KO mice [3]. Therefore, it is difficult to interpret the physiological meaning of the former experimental results. In mouse tumor model, such as colon cancer, IL-27 strengthens anti-tumor activity by supporting production of perforin and granzyme B from CD8^+^ T cells, in addition to the promotion of proliferation and IFN-γ production [5].

On the other hand, immunosuppressive activity of IL-27 has been reported. With regard to B cells, IL-27 has been known to influence on various B cell subsets and suppresses antibody production. IL-27Rα overexpression is reported to suppress antibody production in lupus-prone MRL-*Fas^lpr/lpr^* (MRL/*lpr*) mice [6]. Although, as mentioned above, IL-27 induces the expansion of Th1 cells, IL-27 exerts its immunosuppressive effects by inhibiting the development of Th17 cells and inducing IL-10 production [7]. Recently, IL-27 has been identified as a differentiation factor for the IL-10-producing type 1 regulatory T (Tr1) cells [8,9,10]. We also identified a novel IL-27-induced IL-10 production pathway in CD4^+^ T cells mediated by early growth response gene 2 (Egr-2) and B lymphocyte induced maturation protein-1 (Blimp-1) [11].

This review provides a summary of studies describing recent advances related to the structure and production mechanism of IL-27.

## 2. Structure of Interleukin (IL)-27 and Its Production Mechanism

Ebi3, one of the subunit of IL-27, was first reported in 1996 [12]. At that time, it was speculated that another subunit must exist for Ebi3 secretion, because Ebi3 structurally resembles class I cytokine receptor family without membrane anchoring domain, such as IL-12 p40, and human EBI3 is not efficiently secreted from transfected human B-lymphoma cell line. The other subunit, IL-27p28 was identified by computational research on the basis that it might be the member of four-helix bundle cytokines binding to class I cytokine receptor. IL-27p28 was hardly secreted and did not show any biological activity by itself, implying the importance of its partner molecule. Among some candidate molecules binding to IL-27p28, Ebi3 was found to be the most efficient molecule for secretion of IL-27p28 [1]. This heterodimer cytokine was named as IL-27, followed by the identification of its receptor subunits; IL-27Rα and gp130 [13].

These subunits are independently produced from various types of cells. In immune cells, IL-27 is mainly produced from antigen-presenting cells (APCs). The mRNA expressions of IL-27p28 and Ebi3 are induced in APCs by Toll-like receptor (TLR) stimulation, such as lipopolysaccharide (LPS), CpG, and Poly(I:C). Upon IL-27p28 production, nuclear factor (NF)-κB is important in early induction phase mediated by TLR. In addition, IFN-γ and IFN-α/β can amplify IL-27p28 expression by activating IFN response fragment-1 (IRF-1) and IRF-8 [14]. Myeloid differentiation factor (MyD88)-independent Toll/IL-1R-related domain-containing adaptor-inducing IFN (TRIF) mediated activation of IRF-3 and IRF-4 is also related to efficient IL-27p28 and Ebi3 production [15].

Although the structure and production mechanism of IL-27 have been elucidated, the physiological secretion mechanism has remained to be unclear. IL-27p28 and Ebi3 lack disulfide linkage, which other IL-12 family cytokines, such as IL-12 (p35 and p40) and IL-23 (p19 and p40), have. Also, IL-27p28 can show antagonistic activity via receptor blockade [16,17,18]. Recently, the IL-27p28 subunit has been reported as an independent cytokine, also known as IL-30, that activates signal transduction through gp130 receptor in the absence of the Ebi3 [19]. Further studies are necessary to clarify the precise physiological secretion mechanism of IL-27.

## 3. IL-27 in T Cell Immunity

### 3.1. IL-27 in Type 1 Helper T (Th1) Responses

As we mentioned above, IL-27 was first recognized as a pro-inflammatory cytokine. Although IL-27Rα KO mice seem to have no immunological defect in steady state, the mice are more susceptible to various intracellular pathogens as a consequence of defects in Th1 response. The fact that IL-27 promotes naïve CD4^+^ T cell proliferation and IFN-γ production [1] supports the idea that IL-27 facilitates Th1 response. The mechanism of Th1 induction by IL-27 is dependent on STAT1-mediated T-bet activation, which induces IL-12Rβ2 expression thereby sensitize T cells to Th1 prone signals [2]. Moreover, IL-27 can also induce Th1 differentiation through the adhesion molecules intercellular adhesion molecule (ICAM)-1/lymphocyte function-associated antigen (LFA)-1 interaction in a STAT1-dependent, but T-bet-independent mechanism [20].

On the other hand, with an appreciation that IL-27 suppresses Th2 response, as we discussed below, the reduced Th1 response in IL-27Rα KO mice appears to be a secondary consequence of enhanced Th2 response [21]. Furthermore, during chronic infection with *Leishmania* or *Mycobacterium tuberculosis*, IL-27Rα KO or Ebi3 KO mice are able to develop protective Th1 responses that are comparable to those of wild type (WT) counterparts [22,23,24]. Also, aberrant Th1 responses with *Toxoplasma gondii* infection in IL-27Rα KO mice can be cancelled by depleting CD4^+^ T cells [25]. In addition, IL-27 inhibits IL-2 production from T cells. Villarino *et al.* have shown that IL-2 expression is enhanced in IL-27Rα-deficient T cells and exogenous IL-27 inhibits IL-2 production in WT T cells [26]. This suppression of IL-2 by IL-27 is dependent on suppressor of cytokine signaling (SOCS) 3 [27]. As IL-2 plays important roles in proliferation and survival of Th1 cells, these findings may explain the IL-27-mediated suppression of Th1 immunity. Suppression of Th1 response by IL-27 also can be explained by the induction of anti-inflammatory cytokine, IL-10. IL-27 expands IL-10-producing Th1 cells [9,10]. IL-10-dependent anti-inflammatory effect of IL-27 in Th1-driven model of experimental autoimmune encephalomyelitis (EAE) was reported [9]. It is becoming clear that IL-27-mediated signal has suppressive effect on Th1 response, aside from a role in promoting Th1 response.

### 3.2. IL-27 in Th2 Responses

There are several reports describing exaggerated Th2 response to parasite infection in IL-27Rα KO mice. Following infection with the parasite *Trichuris muris*, IL-27Rα KO mice exhibit accelerated expulsion of larval parasites associated with elevated production of parasite-specific IL-4, IL-5, and IL-13 [21]. As blockade of IL-4 prior to infection with *Lesihmania major* induces normal parasite specific Th1 responses in IL-27Rα KO mice, the susceptibility in these mice is not due to a defect in Th1 immunity, but rather a consequence of accelerated Th2 responses. Ovalubmin (OVA)-induced airway hyper-responsiveness is suppressed by IL-27 administration which results in an inhibition of Th2 cell differentiation [28]. In lupus-prone MRL/*lpr* mice, Th1:Th2 balance shifts to Th2-immunity by IL-27Rα deficiency, resulting in a Th2-mediated immunopathology similar to human membranous glomerulonephritis [29]. One of the molecular mechanisms of the IL-27-mediated suppression of Th2 response is the inhibition of the master regulator of Th2 differentiation, GATA binding protein-3 (GATA-3), which is dependent on STAT1 [28,30].

### 3.3. IL-27 in Th17 Responses

There are several reports demonstrated that IL-27 suppresses Th17 responses [31,32]. IL-27 suppresses IL-17 production from CD4^+^ T cells stimulated with α-CD3, α-CD28, IL-6, and TGF-β3 via mainly STAT1-dependent, partially STAT3-dependent mechanism [33]. Furthermore, during *in vitro* Th17 differentiation experiments, IL-27 inhibits the expression of RAR-related orphan receptor (ROR) α and RORγ, which are transcription factors essential for Th17 development. IL-27 also suppresses production of IL-22, which is important for Th17 effector function [34,35]. One of the other mechanisms by which IL-27 inhibits IL-17 production is mediated by IL-10 production by IL-27 stimulation [36]. Moreover, Hirahara *et al.* showed that IL-27-primed CD4^+^ T cells up-regulate expression of programmed death ligand 1 (PD-L1) in a STAT1-dependent manner. When cocultured with naïve CD4^+^ T cells, IL-27-primed T cells inhibit the differentiation of Th17 cells through a PD-1-PD-L1 interaction, and cotransfer of IL-27-primed T cells suppress EAE [37].

Although IL-27 can inhibit the *de novo* Th17 differentiation, there are some contradictory reports as to whether IL-27 could suppress fully differentiated Th17 cells. For example, IL-27 is able to block IL-17 secretion from effector CD4^+^ or CD8^+^ T cells isolated from the central nervous system (CNS) of infected mice [33]. On the other hand, IL-27 cannot inhibit IL-17 production when the memory cells are isolated from mice with EAE [35]. Recently, it was reported that the balance of IL-23 *vs.* IL-12 and IL-27 can be sensed by *bona fide* Th17 cells. Th17 cells integrate these signals into up-regulation of Blimp-1, providing immunosuppressive activity on Th17 cells by inducing IL-10 production [38]. Thus, although IL-27 might be useful to treat Th17-mediated diseases, precise understanding for the effects of IL-27 on development and maintenance of pathogenic Th17 cells is critical to control inflammatory responses.

### 3.4. IL-27 in Tregs

IL-27 was initially shown to suppress Foxp3^+^ Treg differentiation under Treg-inducing condition (TGF-β1 plus IL-2) [3,39]. Moreover, in experimental oral tolerance model where mice are fed OVA, transferred OVA specific CD4^+^ T cells from IL-27Rα KO mice have increased Foxp3 expression, suggesting that IL-27 could limit Foxp3^+^ Treg populations *in vivo* [3]. However, IL-27- or IL-27Rα-deficiency have no effect on numbers and frequency of Foxp3^+^ Tregs. IL-27 transgenic mice have a severe defect in their capacity to produce IL-2, indicating that Foxp3^+^ Treg suppression by IL-27 may be indirect effect through IL-2 modulation [4]. On the other hand, Hall *et al.* showed that IL-27 reduces the frequency of Foxp3^+^ Tregs *in vitro* [40], but also revealed that IL-27 can expand the number of Foxp3^+^ Treg, indicating that the reduction of the frequency of Tregs may be caused by the expansion of non-Treg T cell populations. These results are consistent with a report that IL-27 mediated survival effect is required for Foxp3^+^ Treg expansion [41]. However, the precise effects of IL-27 on naturally-occurring Tregs (nTregs) remain unclear.

An alternative view on the interaction of IL-27 and Tregs is that IL-27 promotes Treg cell expansion and expression of T-bet and CXCR3 [40]. Th1-like Treg subset develops in response to IFN-γ and is specialized to control Th1 responses. In WT mice infected with pathogens, a population of Treg expressing T-bet and CXCR3 emerges and suppresses effector T cell responses. As the frequency of Treg population is reduced at primary inflammatory sites in IL-27Rα KO mice, IL-27 may confer migratory capacity on Treg cells in the presence of Th1-mediated inflammation. IL-27 signal can be transduced by STAT1 that induces T-bet-mediated expression of CXCR3. During infection with *T. gondii*, *L. major*, or *Salmonella*, IL-27 is required for the generation of T-bet^+^CXCR3^+^ Treg, which produce IL-10 and limit T effector responses.

Recently, IL-10-producing Tr1 cells were reported to play an important role in controlling peripheral immune responses, which is distinct from CD4^+^CD25^+^Foxp3^+^ Treg [42,43]. IL-10 is an anti-inflammatory cytokine with a critical role in limiting immune pathology [44]. IL-10 deficient mice die with spontaneously developed inflammatory bowel disease [45]. The importance of IL-10 induction on T cells by IL-27 has already been shown in many disease models, such as infection with *T. gondii*, *L. major*, or *Salmonella* as we mentioned above, and EAE. The IL-10 induction by IL-27 was reported to be dependent on STAT1, STAT3, and inducible costimulator (ICOS) [10,42]. In addition, IL-21 production mediated by activator protein-1 (AP-1) activation through MAPK pathway is important for the maintenance of IL-10 production by IL-27 [42,46]. Apetoh *et al.* reported that aryl hydrocarbon receptor (AhR) and its partner transcriptional factor c-Maf cooperatively activate transcription of IL-10 and IL-21 under IL-27 stimulation. Recently, we have shown another IL-27-induced IL-10 production pathway mediated by Egr-2 and Blimp-1 via STAT3-dependent mechanism [11], indicating that Egr-2 plays an important role in the induction of IL-10-producing Tregs.

### 3.5. IL-27 in CD8^+^ T Cell Responses

In many reports, it has been shown that IL-27 induces the activation of STAT1-5 and augments the proliferation and the expression of T-bet, IFN-γ, and IL-12Rβ2 on CD8^+^ T cells. In addition, IL-27 promotes cytotoxic T lymphocyte (CTL) responses by inducing the expression of granzyme B and perforin in mouse and human CD8^+^ T cells [5,47,48]. The IL-27-mediated generation of CTL responses is dependent on STAT1 activation, resulting in the induction of T-bet and Eomesdermin (Eomes). Recently, T cell-intrinsic IL-27 signaling was reported to be necessary for the generation of maximal T cell responses to subunit vaccination [49]. In that system, the influence of IL-27 on CD8^+^ T cell expansion, affinity maturation, function, and memory programming is mediated by STAT1 and STAT3. However, they also showed that CD8^+^ T cell responses to *Listeria monocytogens* and vaccinia virus are IL-27 independent, suggesting that the effects of IL-27 on CD8^+^ T cell response are context dependent.

Also, a number of *in vivo* studies exist that implicates IL-27 in the regulation of anti-tumor immunity mediated by CD8^+^ T cells [50,51]. IL-27 overexpression prompts anti-tumor CTL responses in mice associated with increased proliferation, T-bet and IL-12Rβ2 expression, and production of IFN-γ.

### 3.6. IL-27 in Tfh Responses

It was already reported that IL-6 and IL-21 have important roles in the generation and maintenance of follicular helper T (Tfh) cells and germinal center (GC) formation in mice. While IL-21 is produced by activated T cells, IL-6 from DCs induces expression of Bcl-6 [52], an essential transcription factor for Tfh differentiation and IL-21 expression. Bcl-6 also induces CXCR5, which is important for Tfh homing to B-cell zones and GC formation [53]. Although stimulation with IL-27 results in IL-21 expression (mentioned in Section 3.4.) and enhances Tfh survival, IL-27 is not necessary for the differentiation of Tfh cells [54]. However, it was recently reported that dendritic cell-specific intracellular adhesion molecule-3-grabbing non-integrin (DC-SIGN) triggering by fucose as well as fucose-carrying pathogen-associated molecular patterns (PAMPs) from parasites induces IL-27 expression on DCs via IFN-stimulated gene factor 3 (ISGF3), resulting in differentiation of T cells into Bcl-6^+^CXCR5^+^PD-1^hi^Foxp1^lo^ Tfh cells [55]. They speculated that threshold levels of IL-27 and IL-6 expression by DCs are required for efficient Tfh differentiation. Although LPS-stimulated DCs produces substantial amount of IL-6, but low amount of IL-27, resulting in insufficient induction of Tfh cells, fucose-specific DC-SIGN signaling combined with LPS suppresses IL-6 production while enhancing IL-27 production and results in successful Tfh development. Considering the report that shows IL-27Rα overexpression suppresses antibody production [6], further investigation will be required to address the effects of IL-27 on Tfh functions.

## 4. Conclusions

**Figure 1:**
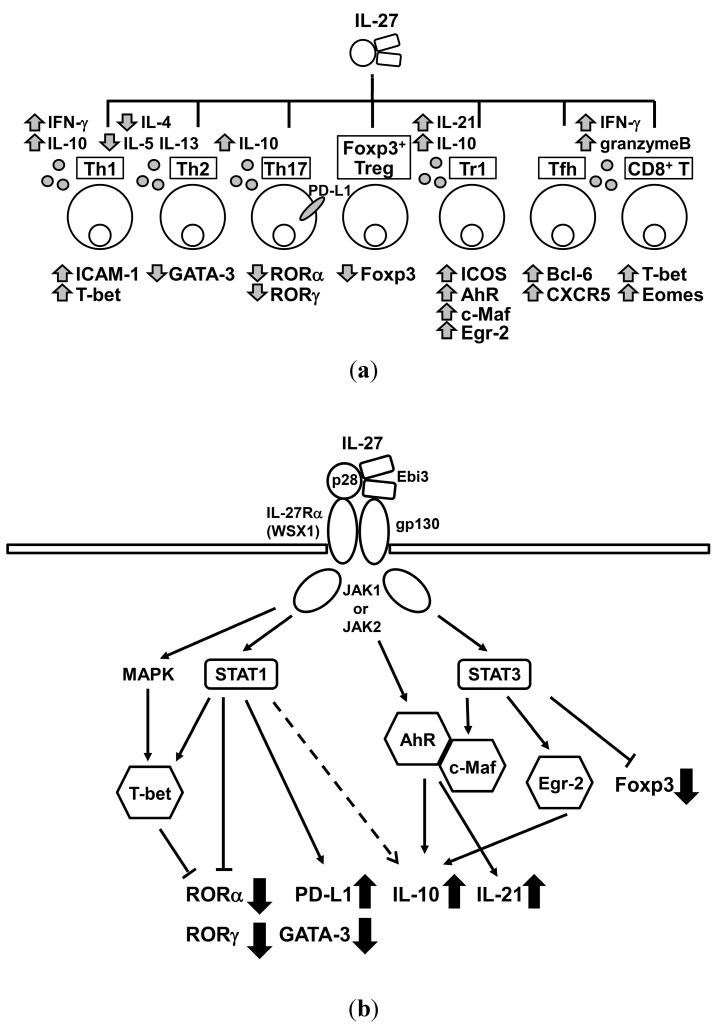
(**a**) Overview of the interleukin (IL)-27 effect on various T cell subsets; and (**b**) IL-27 signal transduction in T cell. Expressions of retinoid-related orphan receptor (ROR)α, RORγ, GATA binding protein-3 (GATA-3), and forkhead box protein P3 (Foxp3) are suppressed by IL-27. IL-10 and programmed death ligand 1(PD-L1) expressions are enhanced. Binding of signal transducer and activator of transcription (STAT) 1 on IL-10 promoter is unclear. IFN-γ, interferon-γ; ICAM-1, intercellular adhesion molecule-1; ICOS, inducible co-stimulator; AhR, aryl hydrocarbon receptor; c-Maf, musculoaponeurotic fibrosarcoma oncogene homolog; Egr-2, early growth response protein 2; Bcl-6, B-cell lymphoma 6; T-bet, T-cell-specific T-box transcription factor; Eomes, eomesodermin; CD8^+^, cluster of differentiation 8 positive; CXCR5, C-X-C chemokine receptor 5; Tr1, type 1 regulatory T; Tfh, follicular helper T; JAK, Janus kinase; MAPK, mitogen-activated protein kinase.

IL-27 effect on T cell immunity is rather complex (Figure 1). Although IL-27 has both pro- and anti-inflammatory effects, as for therapeutic implications of IL-27, many studies exist to show its possibility in controlling diseases, such as bronchial asthma [28], collagen-induced arthritis (CIA) [56,57], EAE [9,35], and tumor progression on the basis of anti-inflammatory effects of IL-27.

Interestingly, human studies, such as on inflammatory bowel diseases and asthma, have described that all the single nucleotide polymorphisms (SNPs) relating with IL-27 have been associated with IL-27p28. It needs to be clarified whether this reflects effects of IL-27 itself or independent role of IL-27p28 subunit. IL27p28, which is independent of Ebi3, was recently shown to have antagonistic effects of gp130-mediated signaling against the activity of IL-6 and IL-27 [16,17]. Additionally, Garbers *et al.* reported that IL-27p28 homodimer or IL-27p28 and soluble IL-6Rα heterodimer can induce signal through gp130 [19]. IL-27p28 is now referred as IL-30. Major questions remain regarding the source of IL-27 and the regulation mechanism of IL-27p28 and Ebi3 *in vivo*. Recently, JAK/STAT inhibitors are promising drugs for treatment of rheumatoid arthritis (RA). Theoretically, these drugs should affect the JAK/STATs utilized by IL-27 and may inhibit the IL-27-mediated anti-inflammatory pathway, however, no adverse inflammatory effects have occurred. This indicates that IL-27-mediated regulatory pathways may be physiologically important for regulating the exacerbation of ongoing inflammation rather than for maintaining continuous immune tolerance.

**Figure 2:**
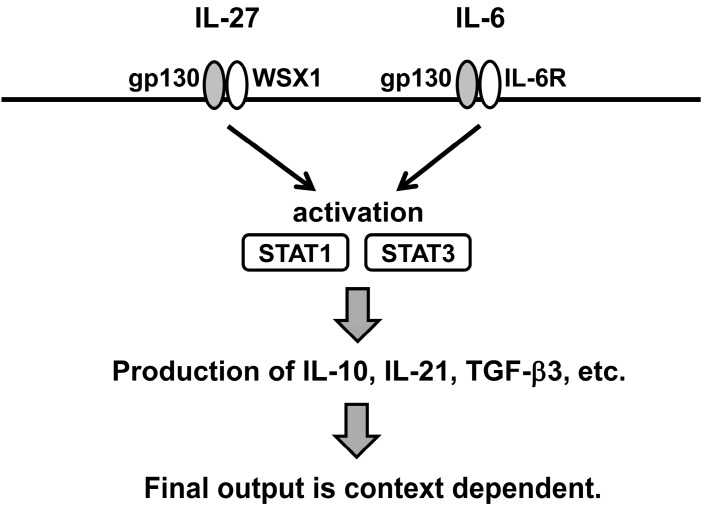
IL-27 and IL-6 can induce common signal transduction pathway in T cell. Output, whether pro- or anti-inflammatory phenotype is granted, may be context dependent.

Although both IL-6 and IL-27 mediate signal transduction through STAT1 and STAT3 activation, its’ effects on Th17 and Treg differentiation are quite different between both cytokines. It suggests that similar signals can bring different transcriptional outputs (Figure 2). IL-6 combined with TGF-β3 was reported to induce more pathogenic Th17 cells compared to IL-6 combined with TGF-β1 [58]. On the other hand, we recently found that TGF-β3 also plays a crucial role in CD4^+^CD25^−^LAG3^+^ Treg (LAG3^+^ Treg) immunosuppressive function on B cells (manuscript in preparation) and found IL-27 induces TGF-β3 on naïve CD4^+^ T cells. Understanding the biology of IL-27 in more detail and revealing the context that determines the outcome are important for applying IL-27 for therapeutic use in various diseases, such as autoimmune disorders, infection, allergies, and cancer.

## References

[1] Pflanz S., Timans J.C., Cheung J., Rosales R., Kanzler H., Gilbert J., Hibbert L., Churakova T., Travis M., Vaisberg E. (2002). IL-27, a heterodimeric cytokine composed of Ebi3 and p28 protein, induces proliferation of naive CD4^+^ T cells. Immunity.

[2] Takeda A., Hamano S., Yamanaka A., Hanada T., Ishibashi T., Mak T.W., Yoshimura A., Yoshida H. (2003). Cutting edge: Role of IL-27/WSX-1 signaling for induction of T-bet through activation of STAT1 during initial Th1 commitment. J. Immunol..

[3] Cox J.H., Kljavin N.M., Ramamoorthi N., Diehl L., Batten M., Ghilardi N. (2011). IL-27 promotes T cell-dependent colitis through multiple mechanisms. J. Exp. Med..

[4] Wojno E.D., Hosken N., Stumhofer J.S., O’Hara A.C., Mauldin E., Fang Q., Turka L.A., Levin S.D., Hunter C.A. (2011). A role for IL-27 in limiting t regulatory cell populations. J. Immunol..

[5] Schneider R., Yaneva T., Beauseigle D., El-Khoury L., Arbour N. (2011). IL-27 increases the proliferation and effector functions of human naive CD8^+^ T lymphocytes and promotes their development into Tc1 cells. Eur. J. Immunol..

[6] Sugiyama N., Nakashima H., Yoshimura T., Sadanaga A., Shimizu S., Masutani K., Igawa T., Akahoshi M., Miyake K., Takeda A. (2008). Amelioration of human lupus-like phenotypes in MRL/*lpr* mice by overexpression of interleukin 27 receptor α (WSX-1). Ann. Rheum. Dis..

[7] Kastelein R.A., Hunter C.A., Cua D.J. (2007). Discovery and biology of IL-23 and IL-27: Related but functionally distinct regulators of inflammation. Annu. Rev. Immunol..

[8] Awasthi A., Carrier Y., Peron J.P., Bettelli E., Kamanaka M., Flavell R.A., Kuchroo V.K., Oukka M., Weiner H.L. (2007). A dominant function for interleukin 27 in generating interleukin 10-producing anti-inflammatory T cells. Nat. Immunol..

[9] Fitzgerald D.C., Zhang G.X., El-Behi M., Fonseca-Kelly Z., Li H., Yu S., Saris C.J., Gran B., Ciric B., Rostami A. (2007). Suppression of autoimmune inflammation of the central nervous system by interleukin 10 secreted by interleukin 27-stimulated T cells. Nat. Immunol..

[10] Stumhofer J.S., Silver J.S., Laurence A., Porrett P.M., Harris T.H., Turka L.A., Ernst M., Saris C.J., O’Shea J.J., Hunter C.A. (2007). Interleukins 27 and 6 induce STAT3-mediated T cell production of interleukin 10. Nat. Immunol..

[11] Iwasaki Y., Fujio K., Okamura T., Yanai A., Sumitomo S., Shoda H., Tamura T., Yoshida H., Charnay P., Yamamoto K. (2013). EGR-2 transcription factor is required for BLIMP-1-mediated IL-10 production in IL-27-stimulated CD4^+^ T cells. Eur. J. Immunol..

[12] Devergne O., Hummel M., Koeppen H., le Beau M.M., Nathanson E.C., Kieff E., Birkenbach M. (1996). A novel interleukin-12 p40-related protein induced by latent Epstein-Barr virus infection in B lymphocytes. J. Virol..

[13] Pflanz S., Hibbert L., Mattson J., Rosales R., Vaisberg E., Bazan J.F., Phillips J.H., McClanahan T.K., de Waal Malefyt R., Kastelein R.A. (2004). WSX-1 and glycoprotein 130 constitute a signal-transducing receptor for IL-27. J. Immunol..

[14] Zhang J., Qian X., Ning H., Yang J., Xiong H., Liu J. (2010). Activation of IL-27 p28 gene transcription by interferon regulatory factor 8 in cooperation with interferon regulatory factor 1. J. Biol. Chem..

[15] Kamiya S., Owaki T., Morishima N., Fukai F., Mizuguchi J., Yoshimoto T. (2004). An indispensable role for STAT1 in IL-27-induced T-bet expression but not proliferation of naive CD4^+^ T cells. J. Immunol..

[16] Shimozato O., Sato A., Kawamura K., Chiyo M., Ma G., Li Q., Tagawa M. (2009). The secreted form of p28 subunit of interleukin (IL)-27 inhibits biological functions of IL-27 and suppresses anti-allogeneic immune responses. Immunology.

[17] Stumhofer J.S., Tait E.D., Quinn W.J., Hosken N., Spudy B., Goenka R., Fielding C.A., O’Hara A.C., Chen Y., Jones M.L. (2010). A role for IL-27p28 as an antagonist of gp130-mediated signaling. Nat. Immunol..

[18] Wang R.X., Yu C.R., Mahdi R.M., Egwuagu C.E. (2012). Novel IL27p28/IL12p40 cytokine suppressed experimental autoimmune uveitis by inhibiting autoreactive Th1/Th17 cells and promoting expansion of regulatory T cells. J. Biol. Chem..

[19] Garbers C., Spudy B., Aparicio-Siegmund S., Waetzig G.H., Sommer J., Holscher C., Rose-John S., Grotzinger J., Lorenzen I., Scheller J. (2013). An interleukin-6 receptor-dependent molecular switch mediates signal transduction of the IL-27 cytokine subunit p28 (IL-30) via a gp130 protein receptor homodimer. J. Biol. Chem..

[20] Owaki T., Asakawa M., Fukai F., Mizuguchi J., Yoshimoto T. (2006). IL-27 induces Th1 differentiation via p38 MAPK/T-bet- and intercellular adhesion molecule-1/LFA-1/ERK1/2-dependent pathways. J. Immunol..

[21] Artis D., Villarino A., Silverman M., He W., Thornton E.M., Mu S., Summer S., Covey T.M., Huang E., Yoshida H. (2004). The IL-27 receptor (WSX-1) is an inhibitor of innate and adaptive elements of type 2 immunity. J. Immunol..

[22] Artis D., Johnson L.M., Joyce K., Saris C., Villarino A., Hunter C.A., Scott P. (2004). Cutting edge: Early IL-4 production governs the requirement for IL-27-WSX-1 signaling in the development of protective Th1 cytokine responses following *Leishmania major* infection. J. Immunol..

[23] Zahn S., Wirtz S., Birkenbach M., Blumberg R.S., Neurath M.F., von Stebut E. (2005). Impaired Th1 responses in mice deficient in Epstein-Barr virus-induced gene 3 and challenged with physiological doses of leishmania major. Eur. J. Immunol..

[24] Holscher C., Holscher A., Ruckerl D., Yoshimoto T., Yoshida H., Mak T., Saris C., Ehlers S. (2005). The IL-27 receptor chain WSX-1 differentially regulates antibacterial immunity and survival during experimental tuberculosis. J. Immunol..

[25] Villarino A., Hibbert L., Lieberman L., Wilson E., Mak T., Yoshida H., Kastelein R.A., Saris C., Hunter C.A. (2003). The IL-27R (WSX-1) is required to suppress T cell hyperactivity during infection. Immunity.

[26] Villarino A.V., Stumhofer J.S., Saris C.J., Kastelein R.A., de Sauvage F.J., Hunter C.A. (2006). IL-27 limits IL-2 production during Th1 differentiation. J. Immunol..

[27] Owaki T., Asakawa M., Kamiya S., Takeda K., Fukai F., Mizuguchi J., Yoshimoto T. (2006). IL-27 suppresses CD28-mediated [correction of medicated] IL-2 production through suppressor of cytokine signaling 3. J. Immunol..

[28] Yoshimoto T., Yasuda K., Mizuguchi J., Nakanishi K. (2007). IL-27 suppresses Th2 cell development and Th2 cytokines production from polarized Th2 cells: A novel therapeutic way for Th2-mediated allergic inflammation. J. Immunol..

[29] Shimizu S., Sugiyama N., Masutani K., Sadanaga A., Miyazaki Y., Inoue Y., Akahoshi M., Katafuchi R., Hirakata H., Harada M. (2005). Membranous glomerulonephritis development with Th2-type immune deviations in MRL/*lpr* mice deficient for IL-27 receptor (WSX-1). J. Immunol..

[30] Lucas S., Ghilardi N., Li J., de Sauvage F.J. (2003). IL-27 regulates IL-12 responsiveness of naive CD4^+^ T cells through STAT1-dependent and -independent mechanisms. Proc. Natl. Acad. Sci. USA.

[31] Villarino A.V., Gallo E., Abbas A.K. (2010). STAT1-activating cytokines limit Th17 responses through both T-bet-dependent and -independent mechanisms. J. Immunol..

[32] Liu H., Rohowsky-Kochan C. (2011). Interleukin-27-mediated suppression of human Th17 cells is associated with activation of STAT1 and suppressor of cytokine signaling protein 1. J. Interferon. Cytokine Res..

[33] Stumhofer J.S., Laurence A., Wilson E.H., Huang E., Tato C.M., Johnson L.M., Villarino A.V., Huang Q., Yoshimura A., Sehy D. (2006). Interleukin 27 negatively regulates the development of interleukin 17-producing T helper cells during chronic inflammation of the central nervous system. Nat. Immunol..

[34] Diveu C., McGeachy M.J., Boniface K., Stumhofer J.S., Sathe M., Joyce-Shaikh B., Chen Y., Tato C.M., McClanahan T.K., de Waal Malefyt R. (2009). IL-27 blocks rorc expression to inhibit lineage commitment of Th17 cells. J. Immunol..

[35] El-behi M., Ciric B., Yu S., Zhang G.X., Fitzgerald D.C., Rostami A. (2009). Differential effect of IL-27 on developing *versus* committed Th17 cells. J. Immunol..

[36] Batten M., Kljavin N.M., Li J., Walter M.J., de Sauvage F.J., Ghilardi N. (2008). Cutting edge: IL-27 is a potent inducer of IL-10 but not FoxP3 in murine T cells. J. Immunol..

[37] Hirahara K., Ghoreschi K., Yang X.P., Takahashi H., Laurence A., Vahedi G., Sciume G., Hall A.O., Dupont C.D., Francisco L.M. (2012). Interleukin-27 priming of T cells controls IL-17 production in *trans* via induction of the ligand PD-L1. Immunity.

[38] Heinemann C., Heink S., Petermann F., Vasanthakumar A., Rothhammer V., Doorduijn E., Mitsdoerffer M., Sie C., Prazeres da Costa O., Buch T. (2014). IL-27 and IL-12 oppose pro-inflammatory IL-23 in CD4^+^ T cells by inducing Blimp1. Nat. Commun..

[39] Huber M., Steinwald V., Guralnik A., Brustle A., Kleemann P., Rosenplanter C., Decker T., Lohoff M. (2008). IL-27 inhibits the development of regulatory T cells via STAT3. Int. Immunol..

[40] Hall A.O., Beiting D.P., Tato C., John B., Oldenhove G., Lombana C.G., Pritchard G.H., Silver J.S., Bouladoux N., Stumhofer J.S. (2012). The cytokines interleukin 27 and interferon-γ promote distinct Treg cell populations required to limit infection-induced pathology. Immunity.

[41] Kim G., Shinnakasu R., Saris C.J., Cheroutre H., Kronenberg M. (2013). A novel role for IL-27 in mediating the survival of activated mouse CD4 T lymphocytes. J. Immunol..

[42] Pot C., Jin H., Awasthi A., Liu S.M., Lai C.Y., Madan R., Sharpe A.H., Karp C.L., Miaw S.C., Ho I.C. (2009). Cutting edge: IL-27 induces the transcription factor c-Maf, cytokine IL-21, and the costimulatory receptor ICOS that coordinately act together to promote differentiation of IL-10-producing Tr1 cells. J. Immunol..

[43] Pot C., Apetoh L., Awasthi A., Kuchroo V.K. (2011). Induction of regulatory Tr1 cells and inhibition of T_H_17 cells by IL-27. Semin. Immunol..

[44] Ouyang W., Rutz S., Crellin N.K., Valdez P.A., Hymowitz S.G. (2011). Regulation and functions of the IL-10 family of cytokines in inflammation and disease. Annu. Rev. Immunol..

[45] Kuhn R., Lohler J., Rennick D., Rajewsky K., Muller W. (1993). Interleukin-10-deficient mice develop chronic enterocolitis. Cell.

[46] Xu J., Yang Y., Qiu G., Lal G., Wu Z., Levy D.E., Ochando J.C., Bromberg J.S., Ding Y. (2009). c-Maf regulates IL-10 expression during Th17 polarization. J. Immunol..

[47] Morishima N., Owaki T., Asakawa M., Kamiya S., Mizuguchi J., Yoshimoto T. (2005). Augmentation of effector CD8^+^ T cell generation with enhanced granzyme B expression by IL-27. J. Immunol..

[48] Morishima N., Mizoguchi I., Okumura M., Chiba Y., Xu M., Shimizu M., Matsui M., Mizuguchi J., Yoshimoto T. (2010). A pivotal role for interleukin 27 in CD8^+^ T cell functions and generation of cytotoxic T lymphocytes. J. Biomed. Biotechnol..

[49] Pennock N.D., Gapin L., Kedl R.M. (2014). IL-27 is required for shaping the magnitude, affinity distribution, and memory of T cells responding to subunit immunization. Proc. Natl. Acad. Sci. USA.

[50] Hisada M., Kamiya S., Fujita K., Belladonna M.L., Aoki T., Koyanagi Y., Mizuguchi J., Yoshimoto T. (2004). Potent antitumor activity of interleukin-27. Cancer Res..

[51] Salcedo R., Hixon J.A., Stauffer J.K., Jalah R., Brooks A.D., Khan T., Dai R.M., Scheetz L., Lincoln E., Back T.C. (2009). Immunologic and therapeutic synergy of IL-27 and IL-2: Enhancement of T cell sensitization, tumor-specific CTL reactivity and complete regression of disseminated neuroblastoma metastases in the liver and bone marrow. J. Immunol..

[52] Nurieva R.I., Chung Y., Hwang D., Yang X.O., Kang H.S., Ma L., Wang Y.H., Watowich S.S., Jetten A.M., Tian Q. (2008). Generation of T follicular helper cells is mediated by interleukin 21 but independent of T helper 1, 2, or 17 cell lineages. Immunity.

[53] Nurieva R.I., Chung Y., Martinez G.J., Yang X.O., Tanaka S., Matskevitch T.D., Wang Y.H., Dong C. (2009). Bcl6 mediates the development of T follicular helper cells. Science.

[54] Batten M., Ramamoorthi N., Kljavin N.M., Ma C.S., Cox J.H., Dengler H.S., Danilenko D.M., Caplazi P., Wong M., Fulcher D.A. (2010). IL-27 supports germinal center function by enhancing IL-21 production and the function of T follicular helper cells. J. Exp. Med..

[55] Gringhuis S.I., Kaptein T.M., Wevers B.A., van der Vlist M., Klaver E.J., van Die I., Vriend L.E., de Jong M.A., Geijtenbeek T.B. (2014). Fucose-based PAMPs prime dendritic cells for follicular T helper cell polarization via DC-SIGN-dependent IL-27 production. Nat. Commun..

[56] Niedbala W., Cai B., Wei X., Patakas A., Leung B.P., McInnes I.B., Liew F.Y. (2008). Interleukin 27 attenuates collagen-induced arthritis. Ann. Rheum. Dis..

[57] Pickens S.R., Chamberlain N.D., Volin M.V., Mandelin A.M., Agrawal H., Matsui M., Yoshimoto T., Shahrara S. (2011). Local expression of interleukin 27 ameliorates collagen-induced arthritis. Arthritis. Rheumatol..

[58] Lee Y., Awasthi A., Yosef N., Quintana F.J., Xiao S., Peters A., Wu C., Kleinewietfeld M., Kunder S., Hafler D.A. (2012). Induction and molecular signature of pathogenic Th17 cells. Nat. Immunol..

